# Anti-inflammatory and pro-anabolic effects of 5-aminosalicylic acid on human inflammatory osteoarthritis models

**DOI:** 10.1016/j.jot.2022.10.003

**Published:** 2022-10-29

**Authors:** Kaihu Li, Yong Zhu, Penghui Zhang, Mauro Alini, Sibylle Grad, Zhen Li

**Affiliations:** a Department of Orthopaedics, Xiangya Hospital of Central South University, Changsha, China; b AO Research Institute Davos, Davos, Switzerland; c Department of Orthopaedic Surgery, The Seventh Affiliated Hospital, Sun Yat-sen University, Shenzhen, China

**Keywords:** Chondrocyte pellet, Inflammatory model, Osteochondral explant, Osteoarthritis, Treatment, 5-aminosalicylic acid

## Abstract

**Background:**

Osteoarthritis (OA) is the most common degenerative joint disease, mainly affecting the elderly worldwide, for which the drug treatment remains a major challenge. Low-grade inflammation plays a pivotal role in OA onset and progression. Exploration of notable anti-inflammatory and disease-modifying drugs on human samples could facilitate the evaluation of therapeutic strategies for OA.

**Methods:**

The anti-inflammatory drug 5-aminosalicylic acid (5-ASA) is a first-line drug for ulcerative colitis (UC), however no study has explored the effects of 5-ASA on articular chondrocytes. In this work, both *in vitro* (chondrocyte pellets) and *ex vivo* (osteochondral explants) human inflammatory OA models were applied to evaluate the effects of 5-ASA.

**Results:**

In the inflammatory pellet model, 5-ASA remarkably downregulated the gene expression of interleukin-6 (*IL-6*), and cyclooxygenase-2 (*COX-2*) while upregulating proteoglycan 4 (*PRG4*) and cartilage oligomeric matrix protein (*COMP*) gene expression. Total glycosaminoglycan (GAG) synthesis by pellets was markedly increased in 5-ASA-treated groups compared with the inflammatory group. In conditioned medium, inflammatory mediators (IL-8, nitric oxide) were markedly inhibited upon 5-ASA treatment. Moreover, histological staining showed 5-ASA retained proteoglycan content and inhibited degradation of extracellular matrix (ECM) core components, aggrecan (ACAN) and collagen type II (COL2). In the inflammatory explant model, 5-ASA mitigated signs of OA development by reducing inflammatory mediators and GAG loss.

**Conclusions:**

These findings suggest that 5-ASA has anti-inflammatory and pro-anabolic effects on human chondrocyte pellet and osteochondral explant inflammatory OA models.

**The translational potential of this article:**

Disease-modifying OA drugs are an unmet clinical need for the treatment of OA. Our study explored and demonstrated the anti-inflammatory and protective effects of 5-ASA on *in vitro* and *ex vivo* human inflammatory OA models, showing its translational potential for OA treatment.

## 1 Introduction

Osteoarthritis (OA) is the most prevalent musculoskeletal degenerative disorder, causing reduced quality of life and increasing economic burden globally [1,2]. OA affects both small (like joints in hands) and large (like knee and hip joints) diarthrodial joints, manifesting recurrent joint pain, swelling, transient morning stiffness, and limited joint motion [1]. As a whole-joint disease, the pathology of OA involves cartilage degeneration, synovitis, and subchondral bone remodeling [1,3]. Among the underlying pathophysiological mechanisms, chronic low-grade inflammation plays a pivotal role in OA onset and progression [3]. Although current widely-used drugs such as analgesics, steroids, and hyaluronic acid exhibit symptom-modifying and anti-inflammatory effects in basic and preclinical studies, none have shown notable clinical success in delaying or halting OA progression [4]. On one hand, these drugs do not possess disease-modifying functions. On the other hand, effective treatment is challenged by the complexity and heterogeneity of OA, in which extensive inflammation- and immunity-related signaling pathways are activated. Thus, there is a substantial clinical need to explore novel drugs which not only target multiple inflammatory pathways in OA development but also have disease-modifying effects for OA treatment.

**Figure 1:**
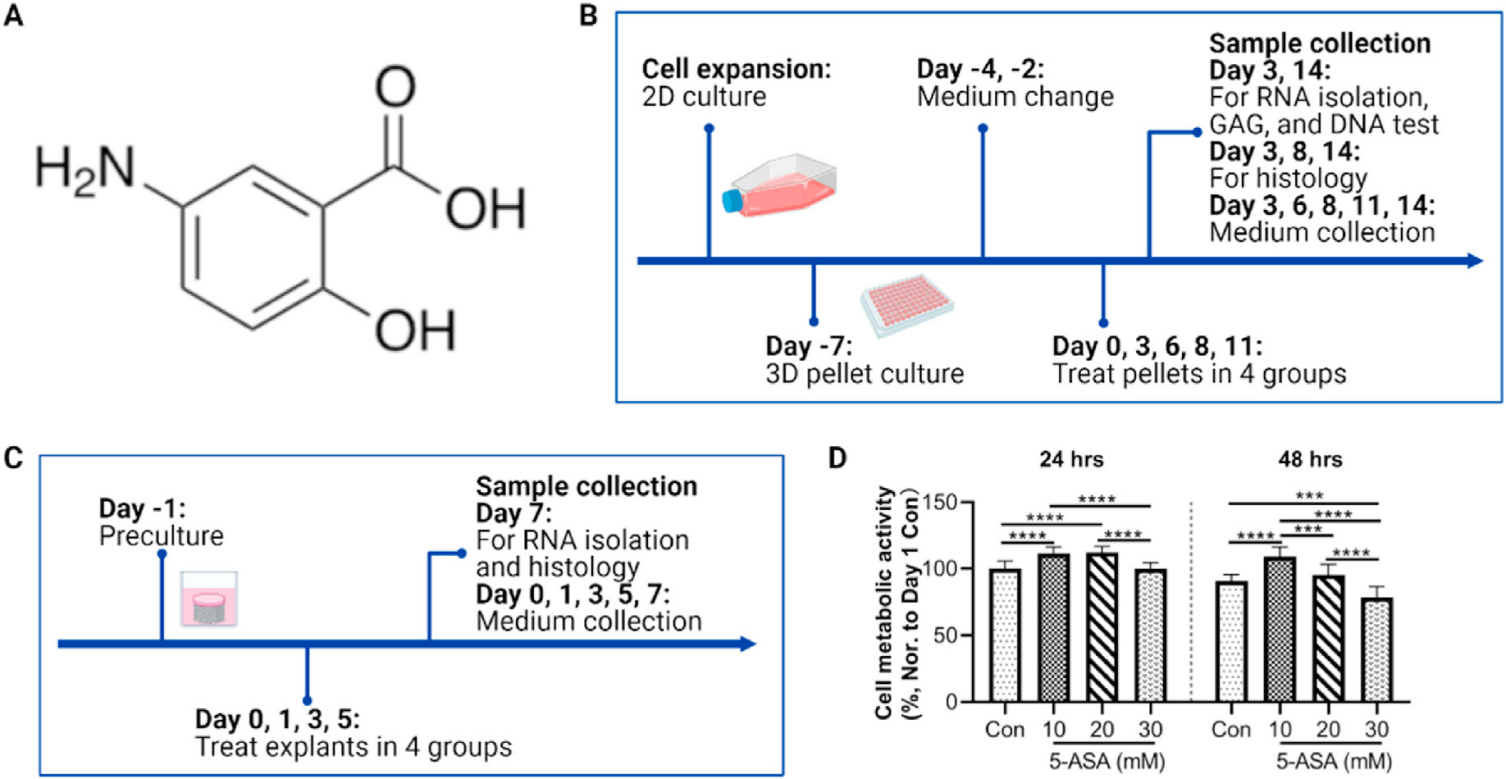
Experimental design and cell viability of chondrocytes treated with 5-ASA. A. Molecular strucutre of 5-ASA. B. Expeimental design of *in vitro* (chondrocyte pellet) inflammatory OA model. C. Experimental design of *ex vivo* (osteochondral explant) inflammatory OA model. D. Cell viability of monolayer-cultured chondrocytes treated with various concentrations of 5-ASA (one way ANOVA, n ​= ​12). Data were presented with mean ​+ SD. ∗∗∗*p* ​< ​0.001, ∗∗∗∗*p* ​< ​0.0001. B and C were made with Biorender.

5-aminosalicylic acid (5-ASA) (Fig. 1A) has been a first-line drug for over thirty years to treat ulcerative colitis (UC), a chronic inflammatory bowel disease affecting the colon [5,6]. UC and OA share some similar pathogenic mechanisms such as activated NF-κB signaling pathway [5,7], increased Toll-like receptor 4 [5,8], tumor necrosis factor-α (TNF-α) [5,9], apoptosis [10,11], and oxidative stress [7,11]. 5-ASA's mechanisms of action to induce UC remission include inhibition of cyclooxygenases, peroxisome proliferator activated receptor γ (PPARγ), NF-κB, and immunosuppressive effects [12,13]. Besides its extensive anti-inflammation efficacy, oral or topical 5-ASA also owns advantages of safety and little side effects after years of formulation development [6].

Hitherto, no studies have evaluated the effects of 5-ASA on OA. The current study aims to investigate the potential of 5-ASA for OA treatment. *In vitro* and *ex vivo* inflammatory OA models with human chondrocytes and osteochondral explants were used to test the effect of 5-ASA in inhibiting inflammation and mitigating OA progression.

## 2 Materials and methods

### 2.1 Reagents and 5-ASA preparation

All the reagents were purchased from Sigma unless otherwise specified. Dulbecco's modified eagle medium high glucose (DMEM HG) was prepared by dissolving 13.38 ​g DMEM HG powder (Gibco), 3.7 ​g sodium bicarbonate, and 0.11 ​g sodium pyruvate in 1 ​L MilliQ water. After filtering through a 0.22 ​μm strainer, DMEM HG was stored at 4 ​°C for further use. 5-ASA was first dissolved in DMEM HG. Then, the pH of 5-ASA in DMEM (around 6.0) was adjusted with 1 ​M NaOH to the same pH as the DMEM HG (around 8.0). After filtering through a 0.22 ​μm strainer, a storage concentration of 5-ASA at 40 ​mM was prepared for further use.

### 2.2 Human chondrocytes isolation and expansion

Cartilage fragments were dissected from osteoarthritic knee or hip joint cartilage of patients undergoing joint replacement operations with donors’ informed consent and ethical approval by cantonal ethical commission (KEK-ZH-NR: 2010-0444/0). Demographic characteristics of included donors were shown in Table 1. Macroscopically non-wear cartilage tissue with intact surface was minced into small pieces and digested in spinner flasks containing DMEM HG, 100 U/mL penicillin plus 100 ​μg/mL streptomycin (1% P/S, PAN™ Biotech), 10% fetal bovine serum (FBS, Seraplus), and 450 U/mL collagenase II (Worthington Biochemical Corporation) for 22 ​h at 37 ​°C and 5% CO_2_. Dissociated cells were cultured in expansion medium, which consisted of DMEM HG, 1% P/S, 10% FBS, 1% nonessential amino acid (NEAA, Gibco), 1 ​ng/mL human transforming growth factor beta 1 (TGF-β1, Fitzgerald), 5 ​ng/mL human fibroblast growth factor basic protein (FGF-2, Fitzgerald) [14]. At their 80% confluency, chondrocytes of passage 0 were detached and preserved in liquid nitrogen. After thawing and expanding, passage 1 to 3 chondrocytes were used in this study.

**Table 1:** Demographic characteristics of patients included.

**Purpose**	**No.**	Gender	Age (years)	Kellgren–Lawrence grade	No. of explants
Chondrocyte isolation	**1**	**Male**	65	2	
	**2**	**Male**	80	4	
	**3**	**Male**	89	4	
	**4**	**Female**	70	3	
Osteochondral explant isolation	**5**	**Male**	60	4	4
	**6**	**Female**	58	3	8
	**7**	**Male**	74	4	4

### 2.3 Human osteochondral explant harvest

Human osteochondral explants were harvested as previously described [15]. Demographic characteristics of included donors were shown in Table 1. Briefly, a trephine with an 8-mm inner diameter was used to extract explants from human femoral heads of patients who underwent hip replacement operation with their informed consent and approval by the cantonal ethical commission (KEK-ZH-NR: 2010-0444/0). Afterwards a circular saw was applied to get explants with 7-mm height. The explants were precultured for 1 day in chondropermissive medium, which contained DMEM HG, 1% P/S, 1% ITS ​+ ​Premix (ITS, Corning), 1% NEAA, and 50 ​μg/mL ascorbic acid (AA).

### 2.4 Cell viability assay

Passage 2 chondrocytes were seeded in 96-well plates with 3000 ​cells per well overnight for cell attachment. The cells were treated in the absence (Control) or presence of 5-ASA at concentrations of 10 ​mM, 20 ​mM and 30 ​mM. After 24-h and 48-h incubation, the medium in each well was replaced with 100 ​μL fresh medium and 20 ​μL CellTiter-Blue Reagent (Promega). After 4-h incubation, fluorescence of each well was measured by microplate reader at excitation wavelength of 560 ​nm and emission wavelength of 590 ​nm. Background fluorescence caused by medium was determined by average readings of wells without cells. Cell viability (%) was normalized to the average value of the Control group after 24-h incubation.

### 2.5 Experimental design

For *in vitro* study (Fig. 1B), passage 3 ​cells were plated in 96-well V bottom plates (Thermo Scientific) by adding 0.25 million cells in each well and 0.25 ​mL chondrogenic medium, which comprised DMEM HG, 1% P/S, 1% ITS, 1% NEAA, 50 ​μg/mL AA, 10 ​ng/mL TGF-β1, 0.1 ​μM dexamethasone (Dex). The plates were then centrifuged at 400 ​G for 5 ​min to obtain chondrocyte micromass. After one-week culture, pellets were randomly divided into 4 groups and supplemented with medium as follows: 1) Control: chondropermissive medium; 2) OA: chondropermissive medium +1 ​ng/mL IL-1β (PeproTech) ​+ ​1 ​ng/mL TNF-α (R&D Systems); 3) 5-ASA 10 ​mM: chondropermissive medium ​+ ​1 ​ng/mL IL-1β ​+ ​1 ​ng/mL TNF-α ​+ ​10 ​mM 5-ASA; 4) 5-ASA 20 ​mM: chondropermissive medium ​+ ​1 ​ng/mL IL-1β ​+ ​1 ​ng/mL TNF-α ​+ ​20 ​mM 5-ASA. Medium was changed every 2–3 days and collected on day 3, 6, 8, 11, and 14 for further analysis. Pellets were harvested on day 3 (short term), 8, and 14 (long term) for gene expression, biochemical, and histological analysis.

For the *ex vivo* study (Fig. 1C), explants after preculture were randomly divided into 4 groups as for the *in vitro* study. According to our previous study [15], 5 ​ng/mL IL-1β in combination with 5 ​ng/mL TNF-α were used to induce the inflammatory OA model. Medium was changed every 1–2 days and collected at every medium change, while explants were collected on day 7.

### 2.6 Gene expression analysis

Total RNA from pellets was isolated using TRI Reagent (Molecular Research Center, MRC). Two Pellets in 1 ​mL TRI Reagent were first homogenized by a tissue lyser. After centrifugation, 0.1 ​mL 1-bromo-3-chloropropane (BCP) was added into the supernatant for phase separation. Aqueous phase was carefully transferred into a fresh tube followed by adding 0.25 ​mL isopropanol and 0.25 ​mL high salt precipitation solution (MRC). After centrifugation, the RNA precipitation was washed with 1 ​mL 75% ethanol for 2 times. Subsequently, RNA was dissolved in RNase-free water after centrifugation and air dried. RNA isolation from cartilage explants, as well as the following reverse transcription and quantitative PCR (qPCR) assay were performed as previously described [15]. Briefly, 0.4 ​μg RNA was reverse-transcribed using Superscript Vilo cDNA Synthesis Kit (Thermo Fisher). qPCR was performed with QuantStudio™ 7 Real-Time PCR System (Applied Biosystems). Relative gene expression levels were calculated using 2 ^−ΔΔCT^ method. The primer and probe sequences of tested genes were listed in Table 2.

**Table 2:** Oligonucleotide primers and probes used for qPCR.

Gene	Primer & Probe	Sequence/Catalogue number
RPLP0	Forward Primer (5’→3′)	TGG GCA AGA ACA CCA TGA TG
	Reverse Primer (5’→3′)	CGG ATA TGA GGC AGC AGT TTC
	Probe (5′FAM/3′TAMRA)	AGG GCA CCT GGA AAA CAA CCC AGC
COL2	Forward Primer (5’→3′)	GGC AAT AGC AGG TTC ACG TAC A
	Reverse Primer (5’→3′)	GAT AAC AGT CTT GCC CCA CTT ACC
	Probe (5′FAM/3′TAMRA)	CCT GAA GGA TGG CTG CAC GAA ACA TAC
COX-2	Forward Primer (5’→3′)	TTG TAC CCT GAC AGG ATT CTA TG
	Reverse Primer (5’→3′)	TGT TTG GAG TGG GTT TCA GAA ATA
	Probe (5′FAM/3′TAMRA)	GAA AAC TGC TCA ACA CCG GAA TTT TTG ACA A
ACAN	Forward Primer (5’→3′)	AGT CCT CAA GCC TCC TGT ACT CA
	Reverse Primer (5’→3′)	CGG GAA GTG GCG GTA ACA
	Probe (5′FAM/3′TAMRA)	CCG GAA TGG AAA CGT GAA TCA GAA TCA ACT
PRG4		Hs00981633_m1
COMP		Hs00164359_m1
IL-6		Hs00174131_m1
IL-8		Hs00174103_m1

*Note:* Primers and probes presented with sequences were self-designed, while others with catalogue numbers were from Applied Biosystems.

### 2.7 Glycosaminoglycan (GAG) measurement

After washing in PBS, pellets were digested in 0.5 ​mg/mL Proteinase K (Roche) overnight at 56 ​°C, then the enzyme was inactivated at 95 ​°C for 10 ​min, and the digest frozen at −20 ​°C for GAG and DNA measurement. GAG content in pellets and conditioned medium were measured as previously described [15] with 1,9-dimethyl-methylene blue (DMMB) as color reagent and chondroitin sulfate as standard. The total GAG synthesis per pellet (GAG_total_) during culture duration comprised GAG release into the medium (GAG_medium_) and GAG content in the pellet (GAG_content_), namely GAG_total_ ​= ​GAG_medium_ ​+ ​GAG_pellet_.

### 2.8 DNA quantification

DNA content in pellets was measured with Bisbenzimide (Hoechst 33258). Calf Thymus DNA (Invitrogen) was used to obtain a standard curve with maximum concentration at 12.5 ​μg/mL. Samples and standards with volume of 40 ​μL in duplicates were pipetted into a 96-well white plate. Subsequently, 160 ​μL Hoechst 33258 dye solution (1 ​μg/mL) was added into each well and incubated for 20 ​min with protection from light. Fluorescence was read at excitation wavelength of 360 ​nm and emission wavelength of 465 ​nm by microplate reader.

### 2.9 Enzyme-linked immunosorbent assay (ELISA)

The content of the inflammatory cytokines interleukin-6 (IL-6) and interleukin-8 (IL-8) in medium was evaluated by ELISA (R&D Systems) according to the producer's instructions.

### 2.10 Nitric oxide assay

Nitric oxide (NO) in medium was measured by Griess Reagent System (Promega Corporation) according to the manufacturer's instructions.

### 2.11 Immunohistochemistry staining

Snap-frozen cryosections (thickness: 10 ​μm for pellets, 8 ​μm for explants) were used for immunohistochemistry (IHC) staining. Samples were fixed in 70% and 100% methanol subsequently for 10 ​min each at room temperature, then air dried overnight. Sections for aggrecan (ACAN) immunostaining were pretreated in 10 ​mM DL-Dithiothreitol (DTT) reduction solution for 2 ​h at 37 ​°C and then 40 ​mM iodoacetamide alkylation solution for 1 ​h at 37 ​°C. After washing in deionized water to remove the cryocompound, all slides were incubated in 0.3% hydrogen peroxide (H_2_O_2_) for 30 ​min to remove endogenous peroxidase. Afterwards, 1.5 U/mL hyaluronidase was used for antigen retrieval of collagen type II (COL2) at 37 ​°C for 30 ​min, while 0.25 U/mL chondroitinase ACII was applied in ACAN epitope retrieval under the same conditions. After treatment with horse serum for 1 ​h, sections were incubated with primary antibodies against COL2 (DSHB, CIICI; 2 ​μg/mL), and ACAN (DSHB, 1C6; 5 ​μg/mL) for 30 ​min at room temperature, successively followed by binding with biotinylated horse anti-mouse secondary antibodies (Vector laboratories, BA-2001; 1:200) for 30 ​min, ABC-complex (Vector laboratories) for 30 ​min, and DAB (Vector laboratories) for 4 ​min. Slides incubated with PBS under the same conditions, instead of primary antibody, were as used as negative control (Neg). Nuclei were stained with Mayer's hematoxylin for 30 ​s. The percentage of positive stained area in the whole section was quantified using Image J software [16]. Briefly, positive-stained area was automatically calculated by setting an optimal threshold conforming to its original figure, while the whole pellet area was circled manually. The percentage of positive stained area was obtained by dividing the positive stained area by the whole pellet area and then multiplying by 100.

### 2.12 Safranin O/Fast Green staining

Sections were fixed with the same method as described above for IHC staining. Slides were then stained with Weigert's hematoxylin for 10 ​min, followed by 0.02% Fast Green for 6 ​min and 0.1% Safranin O for 12 ​min as previously described [15]. The percentage of Safranin O-stained area in pellet section or unstained area in the explant section was quantified using Image J software. For each pellet section, Safranin O-stained area was automatically calculated by setting an optimal threshold, while the whole pellet area was circled manually. The ratio of Safranin O-stained area to its whole pellet area was used for the calculation of percentage value. For explant sections, the percentage of unstained area was assessed as previously described [15].

### 2.13 Statistical analysis

The statistical analysis was performed using GraphPad Prism 8.4.0. Shapiro–Wilk test was first carried out to assess data normality in each group. One-way analysis of variance (ANOVA) was then performed for data with normal distribution, while Kruskal–Wallis test was used for non-normally distributed data. Statistical significance was defined with a p ​< ​0.05.

## 3 Results

### 3.1 Cytotoxicity of 5-ASA on chondrocytes

Cell viability of chondrocytes under stimulation of different 5-ASA concentrations was evaluated by CellTiter-Blue Reagent. After 24-h incubation, 5-ASA showed no toxic effects on chondrocytes, while chondrocytes in 30 ​mM 5-ASA group presented a significant cell viability decrease in comparison to the cells without 5-ASA treatment after 48-h incubation (Fig. 1D). Thus, 5-ASA at concentrations of 10 ​mM and 20 ​mM were applied for further studies.

### 3.2 5-ASA modulates inflammatory and anabolic gene expressions in pellet model

**Figure 2:**
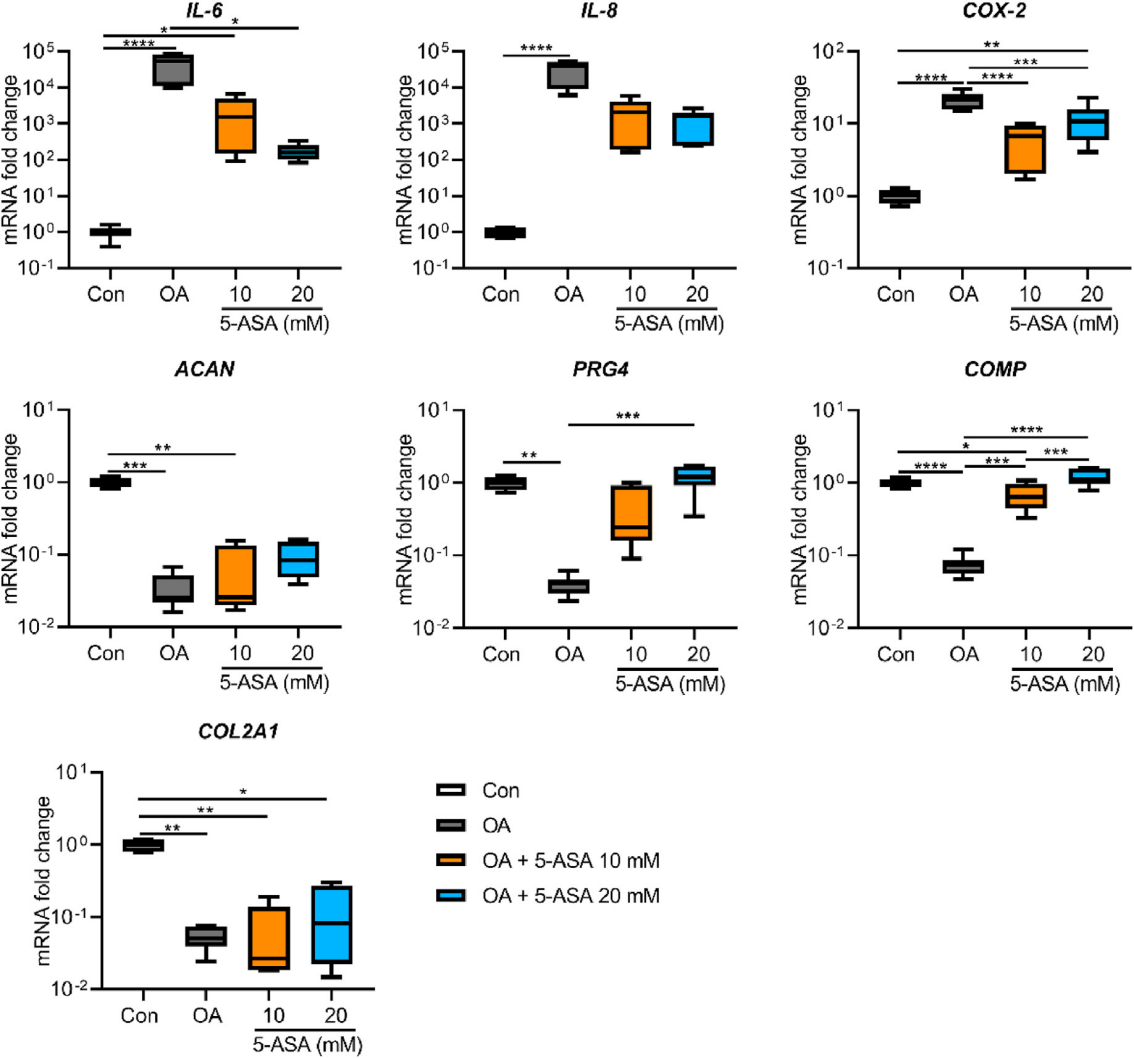
5-ASA regulated anabolism- and inflammation-related gene expression levels of chondrocytes in pellet OA model after 3-day treatment (*COX-2*, *COMP*, one-way ANOVA; *IL-6*, *IL-8*, *ACAN*, *PRG4*, *COL2A1*, Kruskal–Wallis test; n ​= ​7). Data were presented as boxplot; center line, median; box limits, 25th to 75th percentiles; whiskers, min to max. ∗*p* ​< ​0.05, ∗∗*p* ​< ​0.01, ∗∗∗*p* ​< ​0.001, ∗∗∗∗*p* ​< ​0.0001.

To evaluate the early effects of 5-ASA on OA pellets at transcriptional level, pellets were collected after 3-day (short term) treatment. As shown in Fig. 2, inflammatory cytokines induced an OA-like phenotype as indicated by gene expression results, featured by decreased anabolic genes (*ACAN*, *COL2A1*, *PRG4*, *COMP*) and increased inflammatory genes (*IL-6*, *IL-8*, *COX-2*) in OA group in comparison with control group. 5-ASA at 20 ​mM sharply reduced gene expressions of *IL-6* and cyclooxygenase-2 (*COX-2*) compared with OA group. Extracellular matrix (ECM)-related genes proteoglycan 4 (*PRG4*) and cartilage oligomeric matrix protein (*COMP*) were significantly elevated upon 20 ​mM 5-ASA treatment. Although not significant, 20 ​mM 5-ASA showed a trend in down-regulation of *IL-8* and up-regulation of *ACAN* and *COL2A1* gene expression. Moreover, 10 ​mM 5-ASA presented similar regulation trends with weaker effects.

### 3.3 5-ASA attenuates inflammation in conditioned medium in pellet model

**Figure 3:**
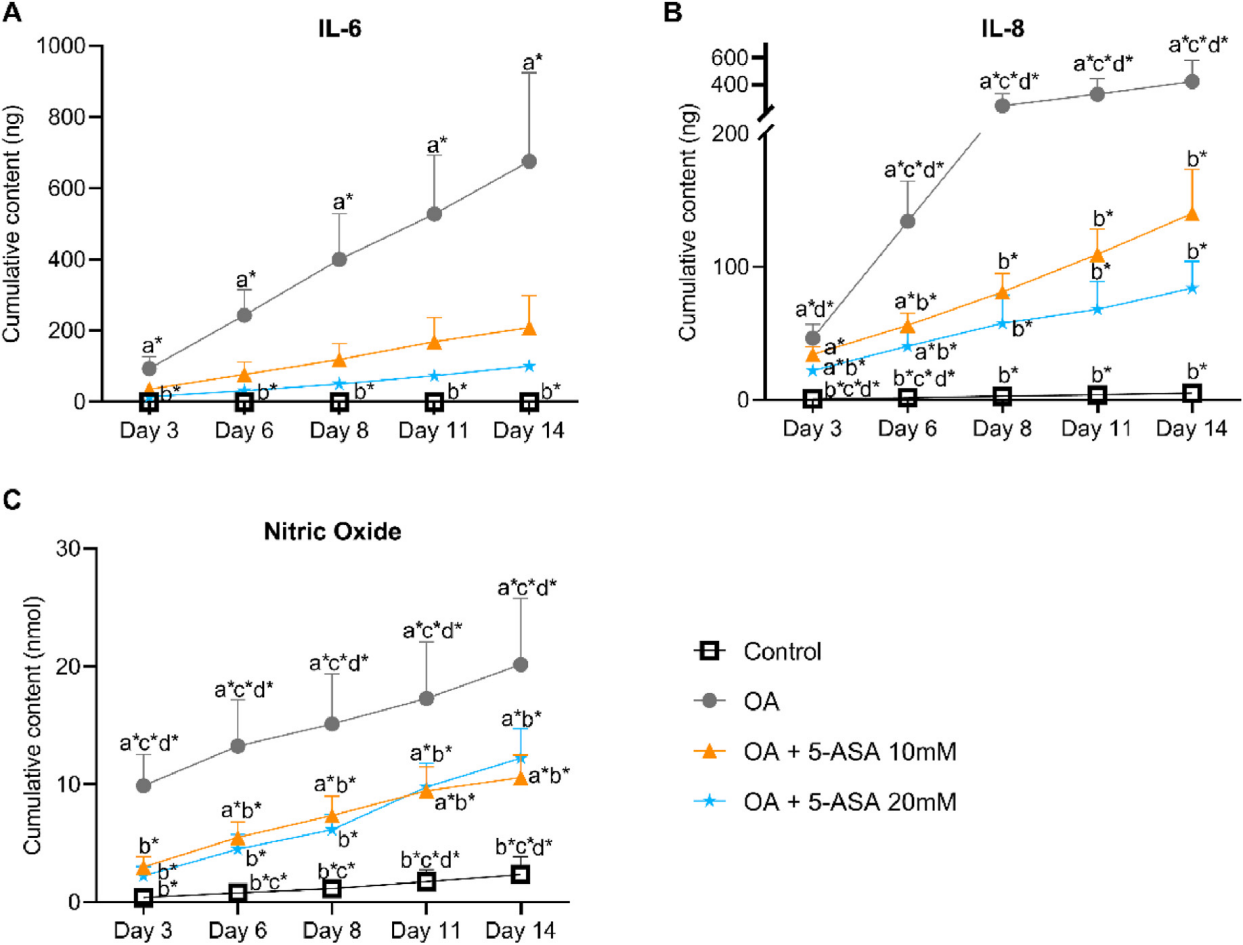
5-ASA inhibited the release of inflammatory markers in pellet OA model. Cumulative content of IL-6 (A), IL-8 (B) and Nitric oxide (NO) (C) in the conditioned medium (IL-6, Kruskal–Wallis test; IL-8, NO, one-way ANOVA; n ​= ​4). Data were presented as mean ​+ SD. a∗, *p* ​< ​0.05 compared with Control group; b∗, *p* ​< ​0.05 compared with OA group; c∗, *p* ​< ​0.05 compared with 5-ASA 10 ​mM group; d∗, *p* ​< ​0.05 compared with 5-ASA 20 ​mM group.

Conditioned medium on day 3, 6, 8, 11, 14 (long term) was collected to analyze released inflammatory mediators. Compared with the control group, cytokines significantly enhanced the release of IL-6, IL-8 and NO into conditioned medium in OA group. Cumulative content of IL-8 and NO in OA group were both markedly decreased by 10 ​mM and 20 ​mM 5-ASA treatment compared with the OA group (Fig. 3B and C). Cumulative IL-6 amount showed a similar trend of reduction in 5-ASA-treated groups although not significantly different from the OA group (Fig. 3A).

### 3.4 5-ASA promotes total GAG synthesis in pellet model

**Figure 4:**
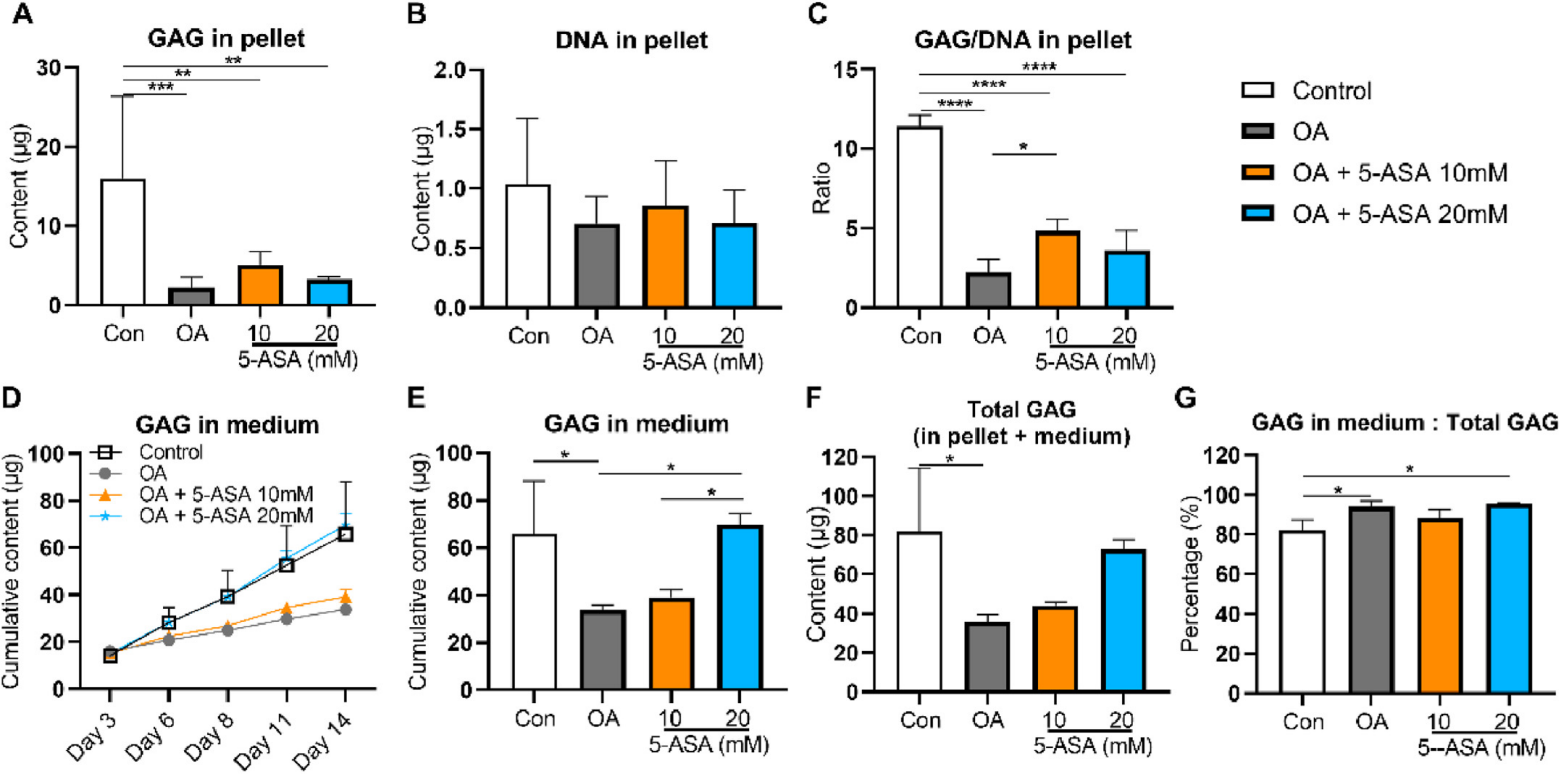
5-ASA promoted GAG synthesis after treatment for 14 days in pellet OA model. GAG content (A), DNA content (B), GAG/DNA (C) per pellet. D, E. Cumulative GAG content released in medium. F. Total GAG synthesis by pellets after 14-day treatment period. G. Percentage of GAG in medium to total GAG synthesis. Data were shown as mean ​+ SD. n ​= ​3. Statistical analysis was performed using one-way ANOVA. ∗*p* ​< ​0.05, ∗∗*p* ​< ​0.01, ∗∗∗*p* ​< ​0.001, ∗∗∗∗*p* ​< ​0.0001.

Pellets were collected on day 14 (long term) to assess total GAG synthesis after long-term treatment with 5-ASA. GAG content per pellet was remarkably reduced in OA group compared with control group (Fig. 4A), while DNA content was comparable among all the groups (Fig. 4B). Compared with the OA group, GAG/DNA per pellet was significantly upregulated in 10 ​mM 5-ASA-treated group and showed a trend of increase in 20 ​mM 5-ASA-treated group (Fig. 4C). Moreover, cumulative GAG release in medium was restored in 20 ​mM 5-ASA-treated group (Fig. 4D and E). According to the formula GAG_total_ ​= ​GAG_medium_ ​+ ​GAG_content_, 5-ASA, especially at 20 ​mM, could promote total GAG synthesis under inflammatory conditions (Fig. 4F). However, the synthesized GAG in 20 ​mM 5-ASA group was mostly released into the conditioned medium (95.49%), but not maintained in the pellet (Fig. 4G).

### 3.5 5-ASA mitigates matrix loss in pellet model

**Figure 5:**
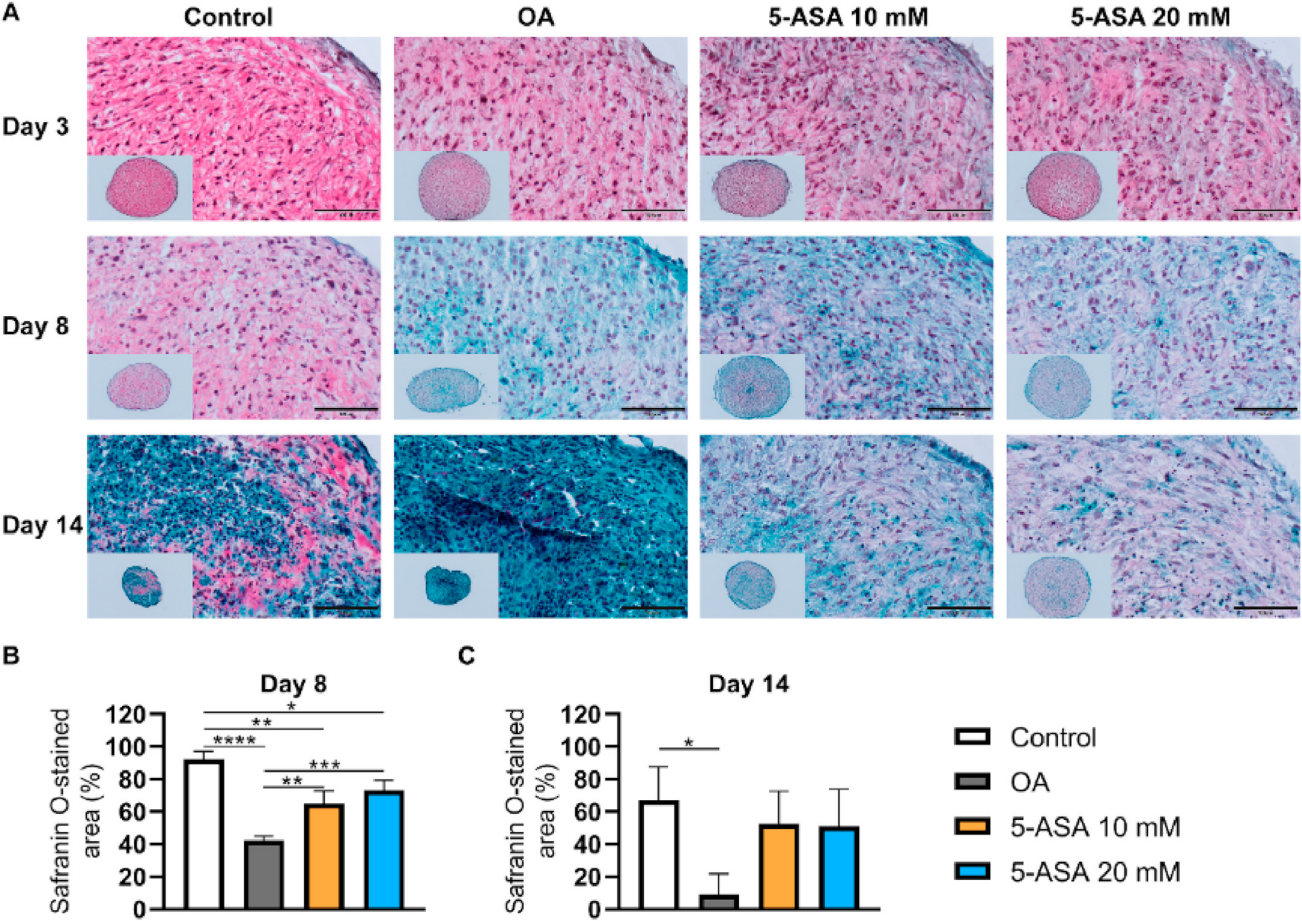
5-ASA preserved PG in pellet OA model. A. Safranin O/Fast Green staining of pellets after 5-ASA treatment for 3, 8, 14 days respectively. Scale bars, 100 ​μm. B, C. Semi-quantitative analysis of Safranin O staining on Day 8 and Day 14 (Day 8, one-way ANOVA; Day 14, Kruskal–Wallis test; n ​= ​3–4). Data were shown as mean ​+ SD. ∗*p* ​< ​0.05, ∗∗*p* ​< ​0.01, ∗∗∗*p* ​< ​0.001, ∗∗∗∗*p* ​< ​0.0001. (For interpretation of the references to color in this figure legend, the reader is referred to the Web version of this article.)

**Figure 6:**
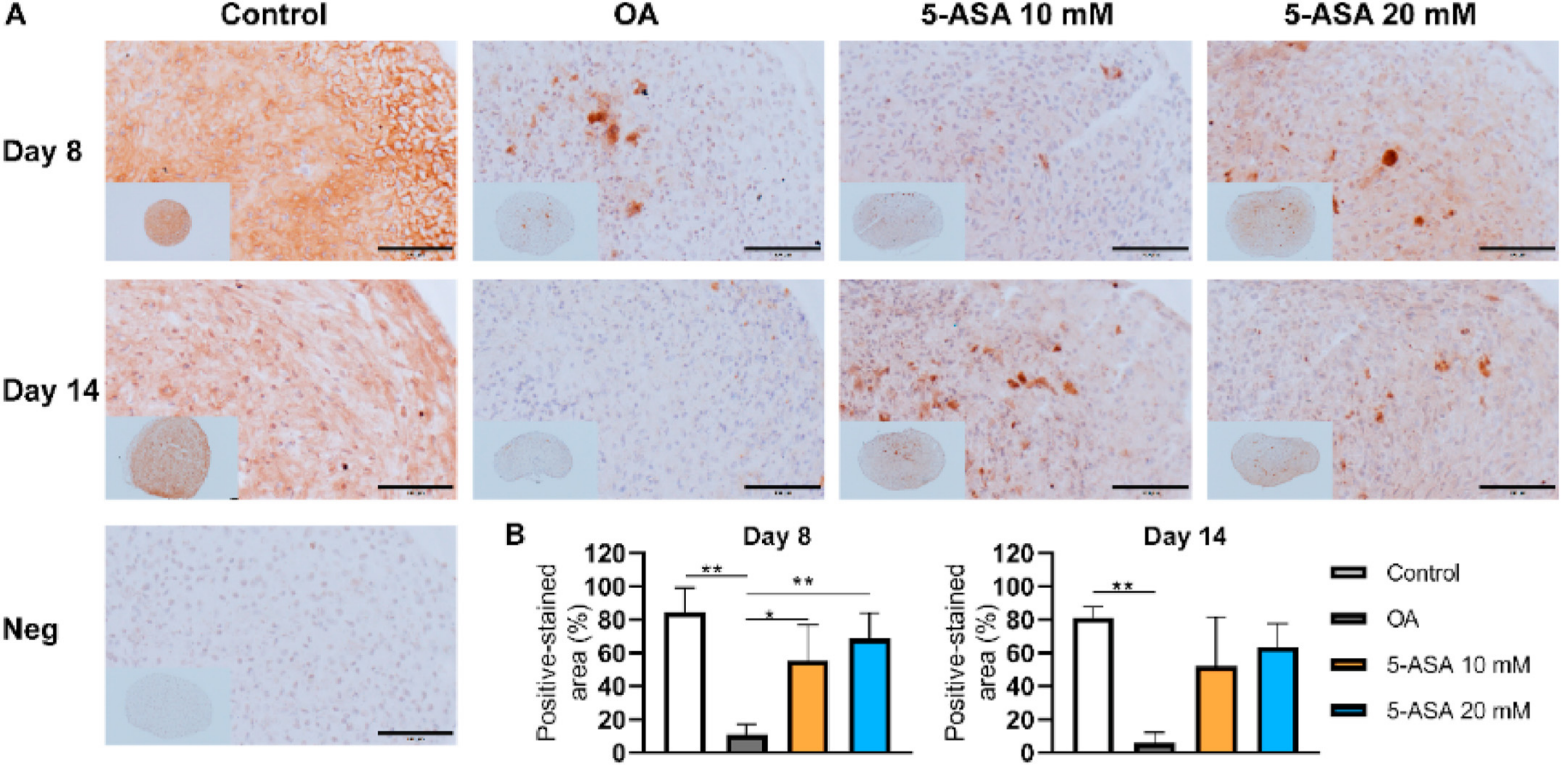
5-ASA reduced aggrecan degradation in pellet OA model. A. Immunohistochemistry staining of ACAN after 5-ASA treatment for 8 and 14 days. Scale bars, 100 ​μm. Neg, negative control. B, C. Semi-quantitative analysis of ACAN immunostaining on Day 8 and Day 14 (Day 8, one-way ANOVA; Day 14, Kruskal–Wallis test; n ​= ​3–4). Data were shown as mean ​+ SD. ∗*p* ​< ​0.05, ∗∗*p* ​< ​0.01.

**Figure 7:**
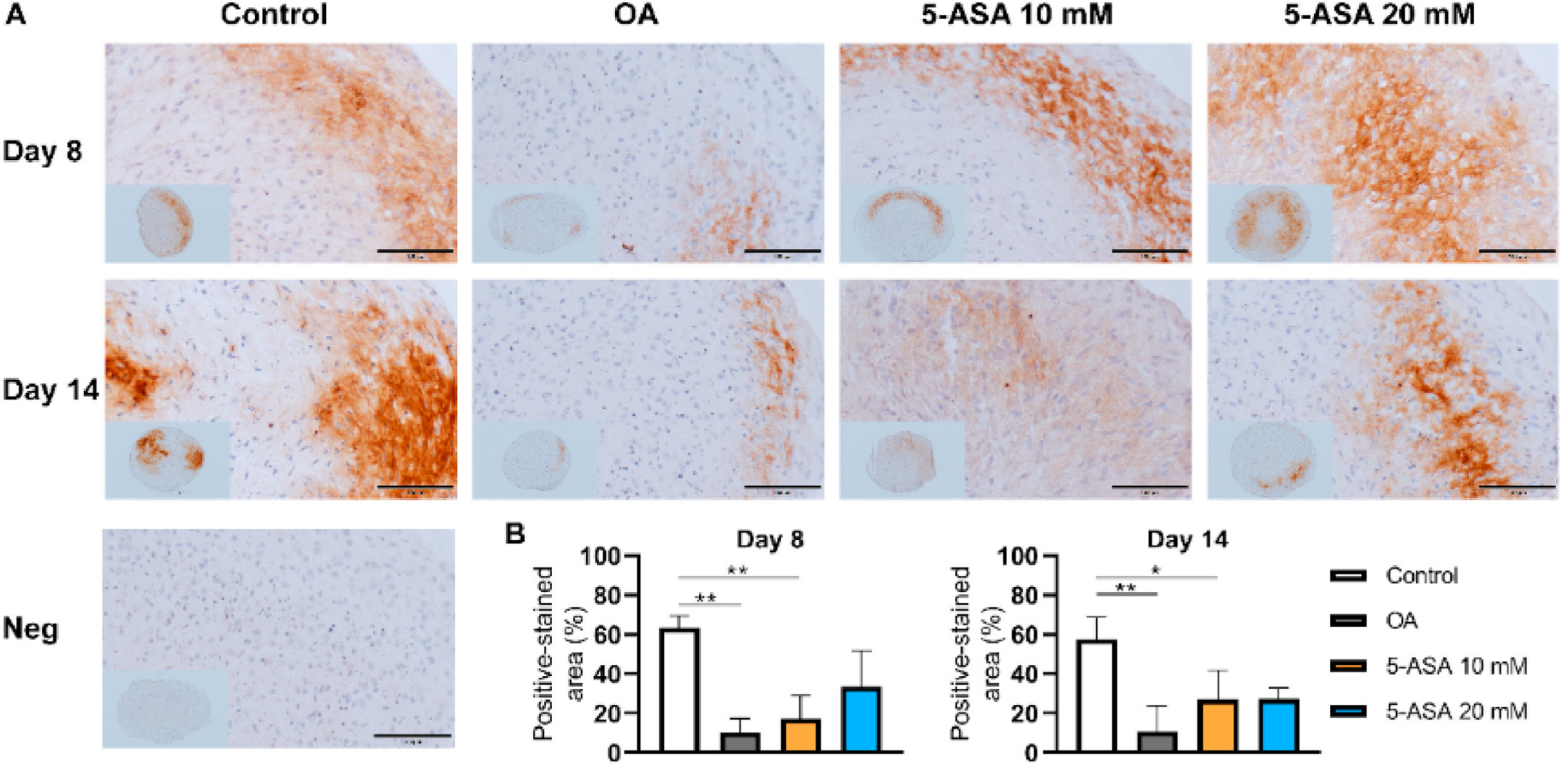
5-ASA reduced collagen type II (COL2) degradation in pellet OA model. A. Immunohistochemistry staining of COL2 after 5-ASA treatment of 8 and 14 days. Scale bars, 100 ​μm. Neg, negative control. B, C. Semi-quantitative analysis of COL2 immunostaining on Day 8 and Day 14 was performed using one-way ANOVA (n ​= ​3–4). Data were shown as mean ​+ SD. ∗*p* ​< ​0.05, ∗∗*p* ​< ​0.01.

Histological staining was carried out to observe the extracellular matrix distribution in pellets. Proteoglycan (PG) staining by Safranin O suggested no evident differences among all groups after 3-day treatment (Fig. 5A). After 8 and 14 days of culture, the OA group showed reduced PG staining compared with the control group, whereas the percentage of PG-stained area in pellets was dramatically increased upon both 10 ​mM and 20 ​mM 5-ASA treatment (Fig. 5A, B, and 5C). IHC staining revealed that ACAN-stained area was restored in both 5-ASA-treated groups after 8 days of culture (Fig. 6A and B), while COL2-stained area was only enhanced in the 20 ​mM 5-ASA group (Fig. 7A and B). These morphology analyses demonstrate a protective role of 5-ASA at protein levels in the pellet inflammatory OA model.

### 3.6 5-ASA inhibits cartilage inflammation and degeneration in explant model

**Figure 8:**
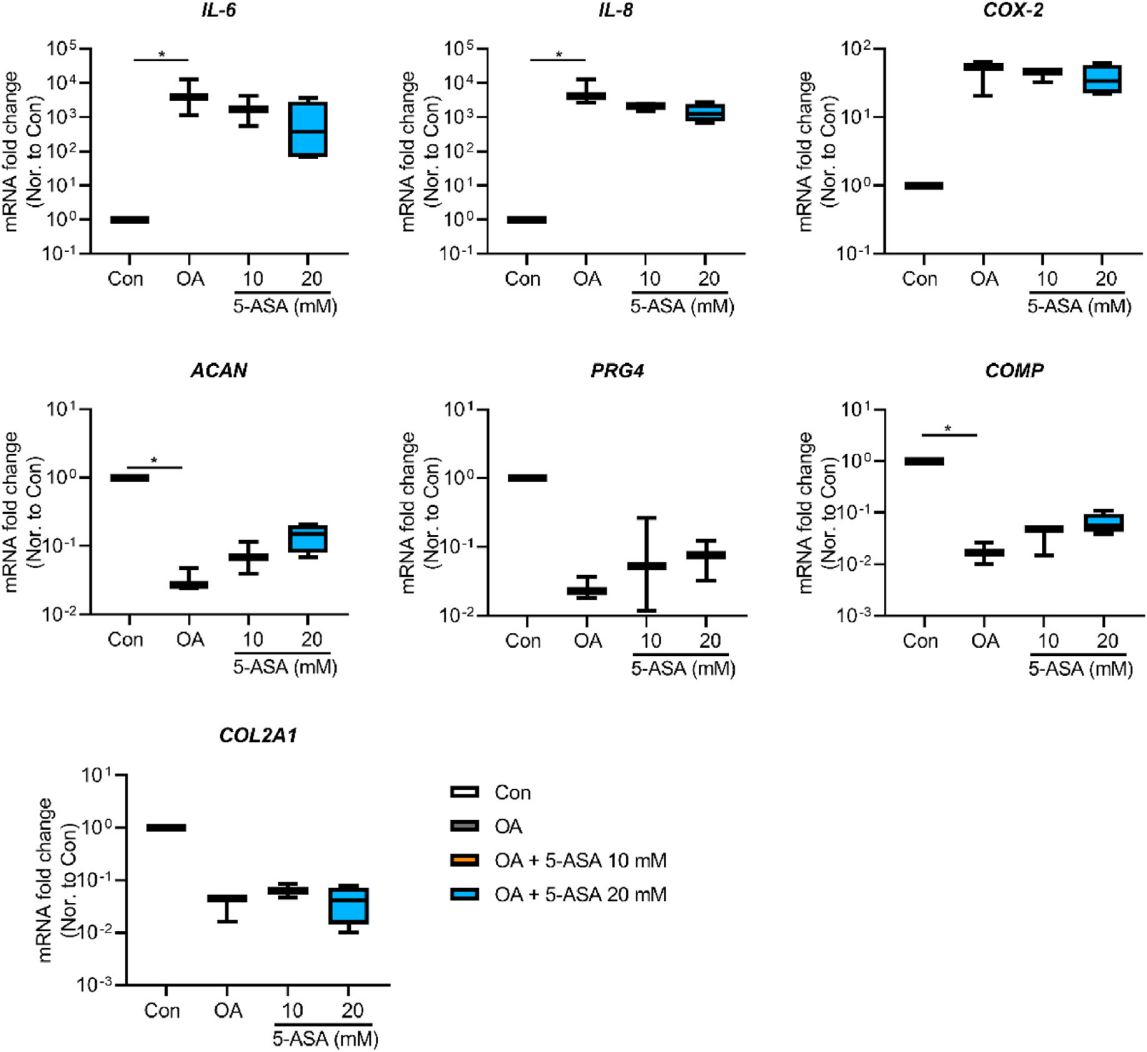
5-ASA upregulated anabolic gene expression and downregualated inflammatory gene expression of chondrocytes in explant OA model after 7 days treatment (n ​= ​3–4). Statistical analysis was performed using Kruskal–Wallis test. Data were presented as boxplot; center line, median; box limits 25th to 75th percentiles; whiskers, min to max. ∗*p* ​< ​0.05.

**Figure 9:**
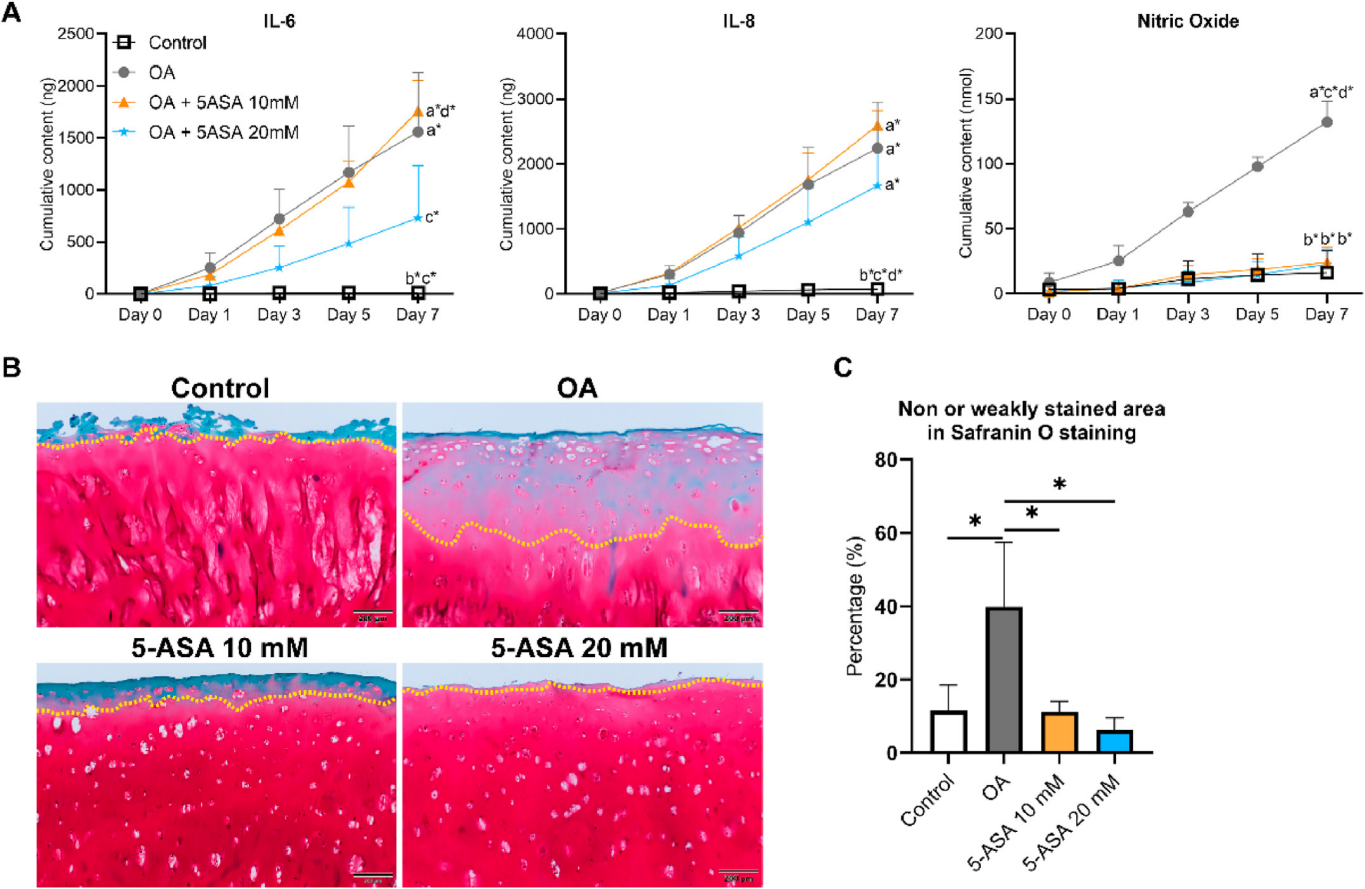
5-ASA attentuated OA progerssion in inflammatory OA model established with human osteochondral explants. A. Cumulative content of IL-6, IL-8, and NO released in conditioned medium (n ​= ​3–4). B. Representative images of Safranin O/Fast Green staining of the cartilage after 7 days. Scale bars, 200 ​μm. Yellow dotted line separates the non- or weakly stained superficial area from the intensely stained deep area in cartilage. C. Semi-quantitative analysis of Safranin O staining (n ​= ​3). Statistical analysis was performed using one-way ANOVA. Data were shown as mean ​+ SD. a∗, *p* ​< ​0.05 compared with Control group; b∗, *p* ​< ​0.05 compared with OA group; c∗, *p* ​< ​0.05 compared with 5-ASA 10 ​mM group; d∗, *p* ​< ​0.05 compared with 5-ASA 20 ​mM group. ∗*p* ​< ​0.05. (For interpretation of the references to color in this figure legend, the reader is referred to the Web version of this article.)

To explore the translational possibility of 5-ASA for clinical practice, our preclinical inflammatory OA model established with human osteochondral explants [15] was used to validate the effect of 5-ASA *ex vivo*. Compared with the control group, explants stimulated with IL-1β and TNF-α showed higher gene expression of *IL-6* and *IL8*, and lower gene expression of *ACAN* and *COMP*. Although not significantly different from the OA group, 5-ASA at 20 ​mM showed a trend of down-regulation on gene expression levels of *IL-6* and *IL-8.* Upregulated *ACAN* and *COMP* gene expression compared with the OA group indicated pro-anabolic effects of 20 ​mM 5-ASA (Fig. 8). Furthermore, inflammation markers of IL-6 and NO in medium were reduced upon treatment with 5-ASA at 20 ​mM (Fig. 9A). Under normal culture conditions proteoglycan depletion in the superficial cartilage zone was comparable in different donors included in our study (Supplementary Fig. 1). Histologically, the percentage of proteoglycan loss in cartilage induced by IL-1β and TNF-α was restored by 5-ASA at both 10 ​mM and 20 ​mM concentrations (Fig. 9B and C). Taken together, these findings suggest 5-ASA could inhibit inflammation and mitigate cartilage degeneration in the explant inflammatory OA model.

## 4 Discussion

With the formulation development and advent of mesalazine, 5-ASA has been a standard treatment for patients with UC for over thirty years [5,6,12]. 5-ASA exerts multifaceted anti-inflammation mechanisms of action in UC treatment. Since OA shares some common pathogenic changes with UC, we explored the effects of 5-ASA in inflammatory chondrocytes and cartilage explants. In most previous studies, only single IL-1β or TNF-α was used to induce inflammatory OA models. As both cytokines are critical mediators controlling cartilage degeneration [9], in this study the combination of IL-1β and TNF-α was applied to induce models closer to the native OA microenvironment [17,18]. Moreover, the use of human-derived chondrocytes and explants could facilitate the translation of 5-ASA into clinical practice.

For clinical practice, various anti-inflammatory drugs have been developed for OA treatment. Oral selective cyclooxygenase 2 inhibitors such as celecoxib are common agents, although they have been associated with considerable gastrointestinal and cardiovascular complications and show no disease modifying effects [1]. Intra-articular corticosteroids and hyaluronic acid could alleviate short-term joint pain presumably because of their anti-inflammatory actions; however, both lack disease modifying efficacy [1]. Emerging biologic agents targeting IL-1β, TNF, β-nerve growth factor (β-NGF) or inhibiting nitrogen oxide have repeatedly revealed disappointing results from clinical trials [19]. These results indicate that blocking only one cytokine may not be sufficient to counteract OA progression [19]. Besides IL-1β and TNF, other cytokines like IL-6 are also appealing targets which have been detected in osteoarthritic cartilage and synovial fluid [20]. In this preclinical study, 5-ASA inhibited multiple targets (COX-2, IL-6, IL-8, and NO) and enhanced the synthesis of several ECM markers (ACAN, COMP, PRG4, COL2) in both inflammatory pellet and explant models, demonstrating its broad and potent effects of anti-inflammation and pro-anabolism.

Under the extensive inflammatory cascades in chondrocytes induced by IL-1β in combination with TNF-α, 5-ASA showed a remarkable downregulation of inflammatory markers in pellet model. Though mean values were reduced in 5-ASA groups in comparison to the OA group, gene expression of *IL-8* and cumulative release of IL-6 only presented a downregulation trend without statistical significance. Large inter-donor variations among the samples from clinical patients may contribute to statistically insignificant comparison. In the pellet model, the expression of matrix molecules like ACAN, PRG4 and COMP were all upregulated upon 5-ASA treatment, which was further supported by Safranin O staining and histological analysis of ACAN. In 5-ASA-treated groups, the ECM protein COL2 only had a regaining trend without statistical significance transcriptionally, while remarkable differences were observed in immunohistochemistry staining. On one hand, the bioprocess of transcription responds more rapidly to extracellular inflammatory stimulation, while protein translation generally represents a delayed reaction. In our study, the tested gene expression levels varied significantly between the groups after 3-day (short term) treatment, while Safranin O staining still showed no differences. On the other hand, mRNA usually represents a transient status, whereas ECM proteins resulted from their cumulative synthesis or breakdown during culture duration. These reasons might cause the discrepancy of ECM markers between mRNA and protein levels.

In our pellet study, GAG content in medium of the control group was much higher than that in the OA group, seemingly contrary to the fact that GAG levels in synovial fluid of OA patients parallel their cartilage degeneration [21]. Unlike authentic native cartilage tissue, chondrocyte pellets consist only of cells prone to form cartilaginous structure. With this purpose, the GAG anabolism in pellets ran at a relatively high level in the control group, while the matrix structure required to retain the synthesized GAG was still not established. Inflammation disrupted this balance predominantly by inhibiting GAG synthesis. Therefore, in the OA group less GAG was released into the medium. However, the ratio of GAG in medium to total GAG synthesis was still higher in the OA group than that in the control group, showing inflammation could impair GAG retention, possibly due to diminished collagen production. Total GAG synthesis, especially upon 20 ​mM 5-ASA treatment, was totally regained to the same level as the control group, suggesting 5-ASA could promote GAG production under inflammatory conditions.

Histological analysis also confirmed the effectiveness of 5-ASA in retaining ECM components. However, the ECM structure in 5-ASA-treated groups was still not comparable to the control group. In this research, we not only applied two classical cytokines simultaneously, but also with multiple repetitions every 2–3 days, leading to constant combined effects that may be stronger compared to previous *in vitro* and *in vivo* studies. Though some potential drugs may be missed out under these harsh inflammatory conditions during preclinical drug screening, our models should facilitate the selection of drugs with superior treatment effects and higher translation potential.

To further investigate its application in human native cartilage tissue, we evaluated the effects of 5-ASA on inflammatory osteochondral explants extracted from human hip joints. Under inflammatory conditions, inflammation- (*IL-6*, *IL-8*) and anabolism-related (*ACAN*, *COMP*) gene expression levels in cartilage showed an improved trend upon 5-ASA treatment. Moreover, 5-ASA decreased upregulated inflammation mediators (IL-6, NO) in medium and maintained proteoglycan staining in cartilage. However, the applicability of 5-ASA still needs to be further evaluated, since only 4 donors were included to date. Side effects of 5-ASA, a therapeutic active agent in sulfasalazine, in humans have been minimized with years of formulation development [6]. Moreover, as a clinically approved first-line drug for mild and moderate UC for years, wide applicability of 5-ASA in heterogeneous populations has been tested [5]. Oral administration of 5-ASA is quite common and suitable for patients with UC, whereas articular cartilage is an avascular and aneural tissue in a joint cavity filled with synovial fluid, which makes oral drugs hard to reach the diarthrodial joint with high concentration. Nevertheless, intra-articular drug injection can be a great alternative to deliver drugs which target cartilage, as is shown by widely-used intra-articular injected sodium hyaluronate and betamethasone for patients with joint disorders. Similarly, it might be preferable to apply 5-ASA by intra-articular injection, instead of oral administration, to increase its concentration in joints and reduce its side effects. Though half-life of the drug in the joint could be tested in an *in vivo* small animal study, the anatomy and biomechanics of cartilage in small animals (thickness 30 ​μm in mice, 100 ​μm in rats) are considered quite different from human's (thickness 2.2 ​mm) [22], sometimes making findings on effective doses not beneficial for clinical translation. Therefore, a cutting-edge controlled drug delivery system may be needed and should be helpful to retain a high concentration of 5-ASA in the joint if it shows a great efficacy [23].

There also exist some limitations in our study. The most effective concentration of 5-ASA varied depending on the assays or markers, which needs more careful consideration about the optimal 5-ASA dose for clinical translation. Though total GAG could be restored by 20 ​mM 5-ASA, the GAG retention could not, demonstrating its limitation in anti-catabolism, which may be compensated by combination drug therapy. Moreover, the underlying mechanism of action of 5-ASA should also be explored in future studies, as well as its retention and penetration in cartilage *in vivo*.

In summary, our findings reveal that 5-ASA has anti-inflammatory and pro-anabolic effects on human *in vitro* and *ex vivo* inflammatory OA models, which may be considered as a promising drug candidate for local OA treatment.

## Authorship

K.H. Li, S. Grad, and Z. Li contributed to the design of the study; K.H. Li, and Z. Li contributed to the acquisition, analysis, and interpretation of the data; K.H. Li drafted the manuscript; Y. Zhu, P.H. Zhang, M. Alini, S. Grad, and Z. Li revised the manuscript critically; and all the authors provided approval for publication of the content.

## Funding

This study was funded by 10.13039/501100001702AO Foundation. Kaihu Li was funded by 10.13039/501100004543China Scholarship Council.

## Declaration of competing interest

The authors declare that they have no known competing financial interests or personal relationships that could have appeared to influence the work reported in this paper.

## References

[1] Martel-Pelletier J., Barr A.J., Cicuttini F.M., Conaghan P.G., Cooper C., Goldring M.B. (2016). Osteoarthritis. Nat Rev Dis Prim.

[2] Safiri S., Kolahi A.A., Smith E., Hill C., Bettampadi D., Mansournia M.A. (2020). Global, regional and national burden of osteoarthritis 1990-2017: a systematic analysis of the Global Burden of Disease Study 2017. Ann Rheum Dis.

[3] Robinson W.H., Lepus C.M., Wang Q., Raghu H., Mao R., Lindstrom T.M. (2016). Low-grade inflammation as a key mediator of the pathogenesis of osteoarthritis. Nat Rev Rheumatol.

[4] Mobasheri A. (2013). The future of osteoarthritis therapeutics: targeted pharmacological therapy. Curr Rheumatol Rep.

[5] Ungaro R., Mehandru S., Allen P.B., Peyrin-Biroulet L., Colombel J.-F. (2017). Ulcerative colitis. Lancet.

[6] Le Berre C., Roda G., Nedeljkovic Protic M., Danese S., Peyrin-Biroulet L. (2020). Modern use of 5-aminosalicylic acid compounds for ulcerative colitis. Expet Opin Biol Ther.

[7] Lepetsos P., Papavassiliou K.A., Papavassiliou A.G. (2019). Redox and NF-kappaB signaling in osteoarthritis. Free Radic Biol Med.

[8] Gomez R., Villalvilla A., Largo R., Gualillo O., Herrero-Beaumont G. (2015). TLR4 signalling in osteoarthritis--finding targets for candidate DMOADs. Nat Rev Rheumatol.

[9] Kapoor M., Martel-Pelletier J., Lajeunesse D., Pelletier J.P., Fahmi H. (2011). Role of proinflammatory cytokines in the pathophysiology of osteoarthritis. Nat Rev Rheumatol.

[10] Hwang H.S., Kim H.A. (2015). Chondrocyte apoptosis in the pathogenesis of osteoarthritis. Int J Mol Sci.

[11] Wan Y., Yang L., Jiang S., Qian D., Duan J. (2022). Excessive apoptosis in ulcerative colitis: crosstalk between apoptosis, ROS, ER stress, and intestinal homeostasis. Inflamm Bowel Dis.

[12] Hauso O., Martinsen T.C., Waldum H. (2015). 5-Aminosalicylic acid, a specific drug for ulcerative colitis. Scand J Gastroenterol.

[13] Kang S., Kim W., Jeong S., Lee Y., Nam J., Lee S. (2017). Oxidized 5-aminosalicylic acid activates Nrf2-HO-1 pathway by covalently binding to Keap1: implication in anti-inflammatory actions of 5-aminosalicylic acid. Free Radic Biol Med.

[14] Francioli S., Cavallo C., Grigolo B., Martin I., Barbero A. (2011). Engineered cartilage maturation regulates cytokine production and interleukin-1beta response. Clin Orthop Relat Res.

[15] Li K., Zhang P., Zhu Y., Alini M., Grad S., Li Z. (2021). Establishment of an ex vivo inflammatory osteoarthritis model with human osteochondral explants. Front Bioeng Biotechnol.

[16] Gutiérrez M.L., Guevara J., Barrera L.A. (2012). Semi-automatic grading system in histologic and immunohistochemistry analysis to evaluate in vitro chondrogenesis. Univ Sci.

[17] Muller S., Acevedo L., Wang X., Karim M.Z., Matta A., Mehrkens A. (2016). Notochordal cell conditioned medium (NCCM) regenerates end-stage human osteoarthritic articular chondrocytes and promotes a healthy phenotype. Arthritis Res Ther.

[18] Ziadlou R., Barbero A., Stoddart M.J., Wirth M., Li Z., Martin I. (2019). Regulation of inflammatory response in human osteoarthritic chondrocytes by novel herbal small molecules. Int J Mol Sci.

[19] Chevalier X., Eymard F., Richette P. (2013). Biologic agents in osteoarthritis: hopes and disappointments. Nat Rev Rheumatol.

[20] Rose-John S., Waetzig G.H., Scheller J., Grotzinger J., Seegert D. (2007). The IL-6/sIL-6R complex as a novel target for therapeutic approaches. Expert Opin Ther Targets.

[21] Kulkarni P., Deshpande S., Koppikar S., Patil S., Ingale D., Harsulkar A. (2016). Glycosaminoglycan measured from synovial fluid serves as a useful indicator for progression of Osteoarthritis and complements Kellgren-Lawrence Score. BBA Clin.

[22] McCoy A.M. (2015). Animal models of osteoarthritis: comparisons and key considerations. Vet Pathol.

[23] Colella F., Garcia J.P., Sorbona M., Lolli A., Antunes B., D'Atri D. (2020). Drug delivery in intervertebral disc degeneration and osteoarthritis: selecting the optimal platform for the delivery of disease-modifying agents. J Contr Release.

