# Supplementary material for: Anti-inflammatory and pro-anabolic effects of 5-aminosalicylic acid on human inflammatory osteoarthritis models
Source: J Orthop Translat. 2022 Oct 29;38:106–16. doi: 10.1016/j.jot.2022.10.003 (PMC9633873; doi:10.1016/j.jot.2022.10.003)
Supplement: Multimedia component 1 [file mmc1.docx]

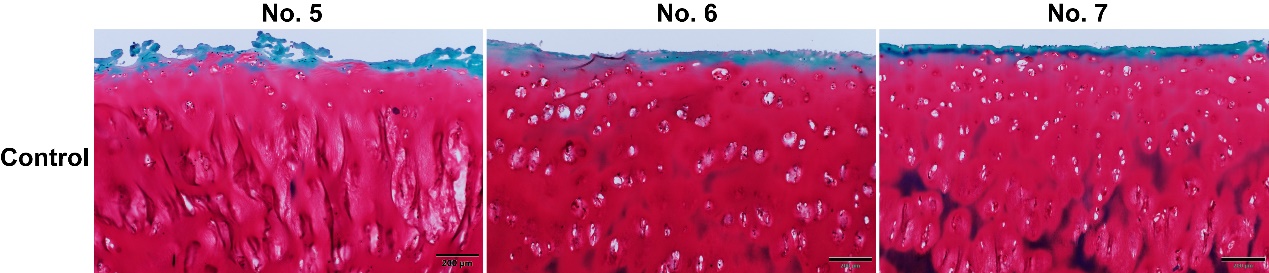


Supplementary Figure 1. Images of Safranin O/Fast Green staining on cartilage tissue in the Control group of patients No. 5, 6, and 7 in osteochodral explant study.
